# The association between migraine and SCORE2 - a Swedish cross-sectional study

**DOI:** 10.3389/fcvm.2026.1854455

**Published:** 2026-10-08

**Authors:** Elsa Regnell, Emelie Stenman, Anton Grundberg, Kristina Sundquist, Beata Borgström Bolmsjö, Veronica Milos Nymberg

**Affiliations:** 1 Center for Primary Health Care Research, Department of Clinical Sciences Malmö, Lund University, Malmö, Sweden; 2 University Clinic Primary Care, Skåne University Hospital, Region Skåne, Sweden

**Keywords:** cardiovascular disease, cross-sectional study, migraine, risk prediction, SCORE2

## Abstract

**Introduction:**

Migraine affects more than one billion people globally and is associated with higher cardiovascular risk. In clinical practice in Europe, cardiovascular risk is often assessed with SCORE2, to identify patients who need preventive care. This study aims to explore the potential association between a register-based diagnosis of migraine and SCORE2 in a cross-sectional study of 40- and 50-year-old people screened for unhealthy lifestyle habits and cardiovascular risk, in Sweden.

**Methods:**

A cross-sectional study was performed with data from a cardiovascular screening program, called Targeted Health Dialogues, which included questionnaires, blood pressure measurements and blood samples. Diagnoses of migraine were collected from a regional patient register, from year 2000 and ahead. Firth's penalized logistic regression was used to calculate odds ratios (ORs) for moderate/high SCORE2 dependent on diagnosis of migraine. The regressions were stratified by sex and stepwise adjusted for age, level of education and country of birth. Multivariable linear regression models with SCORE2 as a continuous variable were also used to assess the association.

**Results:**

Of the 9 190 participants, 875 (9.5%) had a diagnosis of migraine. In 628 cases (72%) it was not possible to distinguish between migraine with and without aura; therefore, these groups were analyzed together. The unadjusted ORs for having a moderate SCORE2 was 0.60 (0.22–1.32) for women with migraine and 1.13 (0.80–1.58) for men with migraine. Adjustments led to almost unaltered results.

**Conclusions:**

In this cross-sectional cohort, the OR of medium/high SCORE2 was not significantly different between individuals with and without a register-based diagnosis of migraine. However, this study could not rule out an existing association. Future studies should include samples that allow for separate analyses of current and past migraine as well as migraine with and without aura.

## 1 Introduction

People with migraine, especially with aura, have been shown to be at higher risk for myocardial infarction and stroke (1). The association remains after adjusting for typical cardiometabolic risk factors, such as BMI, smoking status and diabetes mellitus (2). Migraine affects more than one billion people worldwide (3) and cardiovascular disease (CVD) is the most common cause of death in the world (4), which underscores the importance of studying their association.

In clinical practice, the probability of cardiovascular events is often estimated with a risk score calculator. SCORE2 has been developed by the European Society of Cardiology (5) and is well-established for assessing 10-year risk of CVD in clinical practices in many European countries, including Sweden. To this date, only one study has examined the association between SCORE2 and migraine. That study showed that higher SCORE2 was associated with lower odds of migraine (6), which can be considered unexpected given the association between migraine and higher risk for CVD. The population in that study was Dutch, with a mean age of 44.4 (SD 12.7) years, and the findings need to be tested in other populations to assess generalizability. In Sweden, a standardized cardiovascular screening program for 40- and 50-year-olds called Targeted Health Dialogues (THDs) has been implemented during recent years (7). The aim is to detect CVD risk factors at an early stage, thereby reducing morbidity and mortality. This study aims to examine the association between a register-based diagnosis of migraine and SCORE2 in the 40-and 50-year-old participants in the cardiovascular screening program THDs in Scania, southern Sweden.

## 2 Methods

### 2.1 Study population

Data was collected from THDs, which included extensive questionnaires about the patients’ medical history, heredity factors and lifestyle, as well as blood samples, anthropometric measurements and blood pressure. Our study population was 40- and 50-year-old persons attending THDs between September 20, 2021, and January 10, 2024, in the southernmost region of Sweden: Scania. Everyone who completed a THD was invited to share their data for research, in which case they gave informed written consent and were included in this study. Ethical approval was acquired from the Swedish Ethical Review Authority (registration number 2020-02689, with amendments).

People with diabetes mellitus were excluded, since their cardiovascular risk should be calculated with a different risk score (SCORE2-Diabetes, which requires data points not collected during a THD). SCORE2 is not applicable for people who have already had myocardial infarction or stroke, hence people with such diagnoses were also excluded. These diagnoses were self-reported during the questionnaires preceding the THDs.

### 2.2 Measurements

Levels of plasma lipids were acquired approximately one week prior to each THD, from peripheral venipuncture samples. During the same appointment, blood pressure was measured, using a blood pressure cuff on the upper arm of the participant, who was sitting in a chair and had been resting for at least five minutes. The blood pressure was obtained from both arms at least twice, with at least one minute separating each measurement (8).

To collect information about social background and lifestyle habits, self-reported answers from the THD questionnaires were used. The variables were grouped into categories, as follows.

- Country of birth: Sweden, European countries except for Sweden and countries outside Europe.
- Highest level of education: Elementary school, high school, education after high school <3 years and education after high school ≥3 years.
- Smoking: never smoked, quit smoking >6 months ago, quit smoking <6 months ago, non-daily smoking and daily smoking.

The risk of a cardiovascular event within 10 years was calculated for each study participant, using SCORE2. The risk calculations were based on sex, age, smoking status, systolic blood pressure, total cholesterol, HDL and geographic region (Sweden). SCORE2 was categorized as low, moderate or high, using the same cutoffs as the European Society of Cardiology:

- *Low* SCORE2 was defined as SCORE2 < 2.5% for the 40-year-olds and SCORE2 < 5% for the 50-year-olds.
- *Moderate* SCORE2 was defined as SCORE2 2.5%–7.5% for the 40-year-olds and SCORE2 5%–10% for the 50-year-olds.
- *High* SCORE2 was defined as SCORE2 > 7.5% for 40-year-olds and >10% for 50-year-olds (5).

The diagnoses of migraine were collected from January 1, 2000, to April 21, 2024, from the Patient Administrative Support in Scania (PASiS). The following ICD-10 codes were recorded: G43.0 (migraine without aura), G43.1 (migraine with aura), G43.2 (status migrainosus), G43.3 (complicated migraine), G43.8 other migraine) and G43.9 (migraine, unspecified). In addition, the code G43- was collected. This code comes from the previous diagnosis system used in primary health care in Sweden, KSH97-P, which has gradually been replaced by ICD-10 since January 2011 (9).

### 2.3 Statistical analysis

All continuous variables were presented with mean values and standard deviations, except for SCORE2, which was not normally distributed and instead presented with median and quartile 1 and 3 and as the ordinal categories used in clinical practice, as explained above. Since sex has a large impact on SCORE2 and since migraine is much more common among women, data was stratified by sex.

Based on variable type and group size, appropriate tests were selected to determine if differences in characteristics for patients with and without migraine were statistically significant. Pearson's Chi-Squared test was used for most comparisons where variables were categorical. The exception was when expected cell count was low (<5) and Fisher's exact test was used. The Two Sample t-test was used for all data that were presented as continuous variables, except for SCORE2, where Wilcoxon rank sum test was used instead.

To assess potential associations between SCORE2 and migraine, Firth's penalized logistic regressions were performed with the diagnosis of migraine as the independent variable and SCORE2-group (low or moderate/high) as the dependent variable. This was used to calculate odds ratios (ORs) and confidence intervals of 95%. These ratios were gradually adjusted for age, education and country of birth. To evaluate whether sex was a significant effect modifier, a likelihood ratio test was performed.

In addition, SCORE2 was analyzed as a continuous variable, using multivariable linear regression. SCORE2 was log-transformed for the linear regression models.

Subgroup analyses were performed (Firth’s penalized regression and linear regression in the same way as described above) for people with migraine with aura and people without a diagnosis of migraine.

Only study subjects with complete data for the included variables were analyzed with regression models. A significance level of 0.05 was used for all calculations, which were all based on R version 4.5.3 (10).

## 3 Results

A total of 15 262 persons completed a THD during the specified time in Scania and 9 450 provided informed study consent. After excluding 260 persons due to a diagnosis of diabetes mellitus, stroke or myocardial infarction, 9 190 people were included in the study (Figure 1).

**Figure 1:**
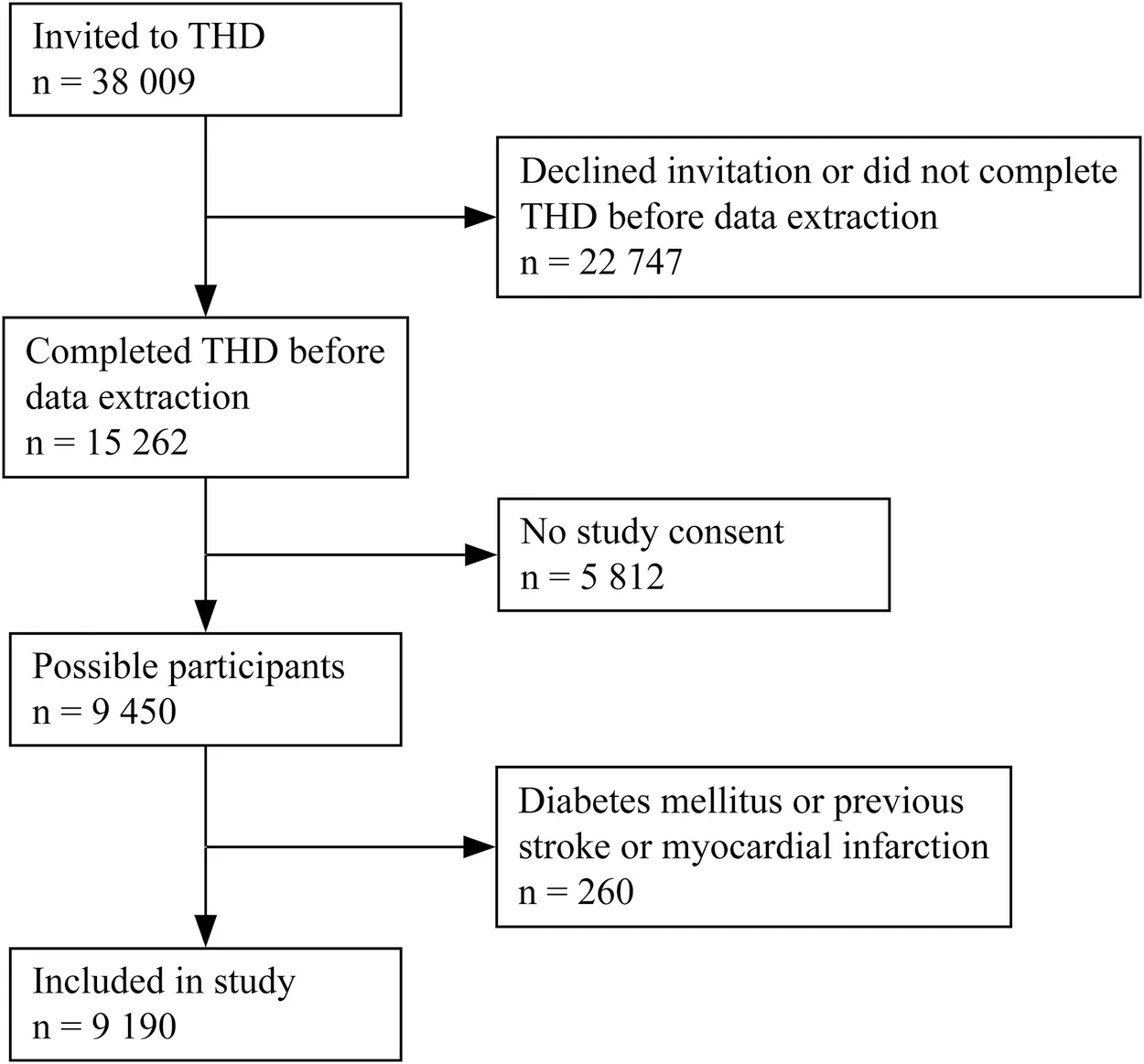
Recruitment of the study cohort. 38 009 persons were invited to a THD in Scania between September 20, 2021, and January 10, 2024. Only people with written study consent were eligible for the study. People with diabetes mellitus or previous stroke or myocardial infarction were excluded. 9 190 persons remained and were part of this study.

6 950 persons (76%) were 40 years old and the remaining 2 240 (24%) 50 years old. 5 070 (55%) were women. In total, 875 (9.5%) had a register-based diagnosis of migraine. Of those with migraine, 687 (79%) were women and 641 (73%) were 40 years old (Table 1). Of those with migraine, 143 (16%) had migraine with aura and 104 (12%) migraine without aura. In 628 cases (72%) it was not possible to determine whether participants with migraine had aura or not.

**Table 1:** Baseline characteristics.

Characteristic	Women			Men		
	No migraine^1^	Migraine^1^	*p*-value^2^	No migraine^1^	Migraine^1^	*p*-value^2^
Age (years)			0.065			>0.90
40	3 333 (76%)	499 (73%)		2 976 (76%)	142 (76%)	
50	1 059 (24%)	188 (27%)		947 (24%)	46 (24%)	
Place of birth			0.074			0.066
Sweden	3 054 (70%)	508 (74%)		2 738 (70%)	144 (77%)	
Europe except for Sweden	569 (13%)	78 (11%)		503 (13%)	14 (7.4%)	
Outside Europe	753 (17%)	100 (15%)		674 (17%)	30 (16%)	
Missing	16	1		8	0	
Highest level of education			0.069			0.20
Education after high school ≥3 years	2 376 (54%)	343 (50%)		1 590 (41%)	64 (34%)	
Education after high school <3 years	758 (17%)	116 (17%)		686 (18%)	30 (16%)	
High school	1 023 (23%)	191 (28%)		1 383 (35%)	81 (43%)	
Elementary or middle school	218 (5.0%)	36 (2.2%)		258 (6.6%)	13 (6.9%)	
Missing	17	1		6	0	

1 n (%).

2 Pearson's Chi-squared test.

The cholesterol levels did not differ significantly between people with and without migraine, except for HDL which was slightly higher for women without migraine. For both women and men, the blood pressure was slightly lower for people without migraine than among those with migraine (the results were significant only for women). A higher proportion of those without migraine had never smoked, compared to people with migraine, but the difference was not significant (Table 2).

**Table 2:** Cardiovascular risk factors - comparison of study subjects with and without migraine. SCORE2 was categorized as low, moderate or high, using the same cutoffs as the European Society of Cardiology: Low SCORE2 was defined as SCORE2 < 2.,5% for the 40-year-olds and SCORE2 < 5% for the 50-year-olds. Moderate SCORE2 was defined as SCORE2 2.5–7.5% for the 40-year-olds and SCORE2 5–10% for the 50-year-olds. High SCORE2 was defined as SCORE2 > 7.5% for the 40-year-olds and >10% for the 50-year-olds.

Characteristic	Women			Men		
	No migraine^1^	Migraine^1^	*p*-value^2^	No migraine^1^	Migraine^1^	*p*-value^3^
Total cholesterol (mmol/l)			0.070			0.3
Mean (SD)	4.7 (0.8)	4.7 (0.8)		5.0 (0.9)	4.9 (1.0)	
Missing	22	4		30	1	
HDL (mmol/l)			0.022			0.3
Mean (SD)	1.6 (0.5)	1.5 (0.4)		1.3 (0.5)	1.3 (0.5)	
Missing	20	4		32	0	
Systolic blood pressure (mmHg)			0.015			0.12
Mean (SD)	119 (14)	120 (14)		129 (14)	131 (14)	
Missing	12	4		12	0	
Diastolic blood pressure (mmHg)			<0.001			0.079
Mean (SD)	78 (10)	79 (10)		82 (10)	83 (10)	
Missing	12	4		13	0	
Self-reported smoking frequency			0.064			0.13
Never smoked	2 938 (67%)	434 (63%)		2 433 (62%)	110 (59%)	
Quit smoking >6 months ago	951 (22%)	181 (26%)		925 (24%)	56 (30%)	
Quit smoking <6 months ago	39 (0.9%)	3 (0.4%)		63 (1.6%)	0 (0%)	
Non-daily smoking	166 (3.8%)	23 (3.4%)		182 (4.6%)	6 (3.2%)	
Daily smoking	290 (6.6%)	45 (6.6%)		313 (8.0%)	15 (8.0%)	
Missing	8	1		7	1	
SCORE2, grouped			0.3			0.6
Low	4 301 (99%)	675 (99%)		3 008 (77%)	141 (75%)	
Moderate	58 (1.3%)	5 (0.7%)		854 (22%)	46 (25%)	
High	0 (0%)	0 (0%)		20 (0.5%)	0 (0%)	
Missing	33	7		41	1	
SCORE2			<0.001			0.13
Median (Q1, Q3)	0.72 (1.53, 1.18)	0.79 (0.55, 1.38)		2.14 (1.54, 3.19)	2.28 (1.66, 3.41)	
Missing	33	7		41	1	

1 n (%).

2 Two Sample t-test; Pearson's Chi-squared test; Fisher's exact test; Wilcoxon rank sum test.

3 Two Sample t-test; Fisher's exact test; Wilcoxon rank sum test.

### 3.1 Firth's logistic regression

The unadjusted OR of having a moderate SCORE2 risk was 0.60 (0.22–1.32) for women with migraine. When adjusted for age, education and country of birth, the results remained largely unchanged [0.58 (0.21–1.28)]. For men with migraine, OR for moderate/high SCORE2 was 1.13 (0.80–1.58). Adjustments had minimal impact on the OR (Table 3). The number of participants in each category can be found in Supplementary Table S1.

**Table 3:** The association between migraine and SCORE2, shown as odds ratios (ORs) for having moderate/high SCORE2 depending on diagnosis of migraine, with 95% confidence intervals. Model 1: Unadjusted. Model 2: Adjusted for age. Model 3: Adjusted for age and highest level of education. Model 4: Adjusted for age, education and country of birth. *Education after high school >3 years **Education after high school <3 years ***Elementary or middle School.

Sex	Variable	OR	Model 1	Model 2	Model 3	Model 4
Women	Migraine status	No migraine (ref.)	1.00	1.00	1.00	1.00
		Migraine	0.60 (0.22–1.32)	0.59 (0.22–1.31)	0.57 (0.21–1.26)	0.58 (0.21–1.28)
	Age	40 years (ref.)		1.00	1.00	1.00
		50 years		1.02 0.97–1.08)	1.01 (0.96–1.07)	1.02 (0.96–1.07)
	Education	* (ref.)			1.00	1.00
		**			3.84 (1.86–8.04)	3.97 (1.92–8.33)
		High school			4.15 (2.15–8.33)	4.09 (2.12–8.23)
		***			7.79 (3.26–17.92)	8.81 (3.49–21.43)
	Country of birth	Sweden (ref.)				1.00
		Europe, except for Sweden				2.31 (1.30–4.31)
		Outside Europe				0.83 (0.37–1.70)
Men	Migraine status	No migraine (ref.)	1.00	1.00	1.00	1.00
		Migraine	1.13 (0.80–1.58)	1.13 (0.80–1.58)	1.08 (0.76–1.52)	1.11 (0.78–1.56)
	Age	40 years (ref.)		1.00	1.00	1.00
		50 years		0.99 (0.98–1.01)	0.99 (0.97–1.01)	0.99 (0.98–1.01)
	Education	* (ref.)			1.00	1.00
		**			1.30 (1.04–1.62)	1.33 (1.06–1.66)
		High school			1.79 (1.50–2.13)	1.84 (1.55–2.20)
		***			3.22 (2.44–4.24)	2.69 (2.02–3.57)
	Country of birth	Sweden (ref.)				1.00
		Europe, except for Sweden				1.39 (1.11–1.73)
		Outside Europe				1.80 (1.48–2.18)

The ORs for migraine with aura was 1.81 (0.37–5.38) for women and 1.27 (0.59–2.5) for men, in the unadjusted model (Supplementary Table S2, with numbers in each model specified in Supplementary Table S3).

Because somewhat opposite patterns appeared between women and men, we compared the models with sex as a potential effect modifier to a model without any effect modifier. The likelihood ratio test for the interaction resulted in a *p*-value of 0.18.

### 3.2 Linear regression

From the linear regression models the coefficient for migraine for women was 0.081 with *p*-value 0.0011 in the unadjusted model. However, the adjusted coefficient of determination (adjusted R2) was very low (0.0019), denoting that migraine had a minimal impact on the model. For men, none of the coefficients of migraine were statistically significant (Table 4). The number of participants in each category can be found in Supplementary Table S4.

**Table 4:** Summary of the linear regression models. Model 1: Unadjusted. Model 2: Adjusted for age. Model 3: Adjusted for age and education. Model 4: Adjusted for age, education and country of birth. *For the variable of migraine.

Sex	Model	Coefficient*	*p*-value*	Adjusted R^2^
Women	1	0.081	0.0011	0.0019
	2	0.053	0.0061	0.40
	3	0.045	0.018	0.42
	4	0.046	0.015	0.42
Men	1	0.042	0.31	<0.001
	2	0.038	0.27	0.29
	3	0.030	0.38	0.31
	4	0.036	0.29	0.32

The coefficients for linear regression models for people with migraine with aura was 0.037 for women and 0.15 for men, both with R^2^ < 0.001 in the unadjusted models (Supplementary Table S5, with number of participants for each model specified in Supplementary Table S6).

## 4 Discussion

We performed two different analyses of the association between migraine and SCORE2 to reduce the potential bias due to a relatively small sample size and limited number of events. The results from the Firth’s penalized logistic regression showed no statistically significant association between migraine and SCORE2. Among women, the linear regression models showed statistically significant associations. However, the regression coefficients for migraine and the adjusted R2 values across all age-stratified models were small, suggesting that the explanatory value and clinical relevance of these associations may be limited. In our study, adjusting for age had minimal effect on the OR for SCORE2 based on the diagnosis of migraine. A major reason is probably that age dictates the conversion from the continuous variable of SCORE2 to a categorical value. The most relevant example is that the cutoff for “low SCORE2” is <2.5% for people <50 years and 5% for people 50–69 years old (5).

The subgroup analyses for migraine with aura should be interpreted with caution due to sparse data. Only two women and ten men who were categorized as having migraine with aura had moderate/high SCORE2, resulting in wide confidence intervals when using Firth's penalized logistic regression.

To our knowledge, there is only one other study examining SCORE2 and the association with migraine and it was performed by Al-Hassany et al. It showed that higher SCORE2 was associated with lower odds for migraine (6), i.e., a similar pattern as among the women in our study, but with migraine as the dependent variable. We chose to study the association between migraine and SCORE2 (inversed dependent and independent variables compared to Al-Hassany et al), since early detection of cardiovascular risk factors and prevention of cardiovascular disease are the main purposes of the Swedish THD programme. It is also more clinically relevant considering that the highest age-standardized incidence for migraine is among 10–14-year-olds (3). Thus, most people are diagnosed with migraine many years before they might develop a cardiovascular disease and increased knowledge of their cardiovascular risk could be useful to prevent cardiovascular outcomes. Another difference between our study and the beforementioned study is that Al-Hassany et al. did not present adjusted values. Instead, they presented ORs stratified by sex and age. The association between lower SCORE2 and migraine is expected in unadjusted models, since SCORE2 increases with higher age and male sex, whereas female sex and younger age are associated with migraine (3, 5).

Other studies examining the relationship between migraine and risk score calculators used the Framingham Risk Score calculator (FRS), which was created based on a population in Massachusetts, USA (11–13). Several studies have shown an association between higher FRS and migraine, even when accounting for age and sex (14–16). These findings are consistent with previous studies showing that migraine, especially with aura, is associated with higher cardiovascular risk.

Another study compared risk prediction based on different risk score calculators, in a global sample of 86 000 people without a history of CVD. 43% of individuals were categorized as being at moderate or high risk using FRS, compared to 28% of the same sample when using SCORE2 (17). Similar results were found in a study where risk score calculators were used to approximate vascular age (where younger vascular than chronological age indicates a healthier circulatory system than the average of the same chronological age). In a sample of 141 persons in Hungary, the vascular age was higher when calculated from FRS compared to from SCORE2 (18). Since risk score calculators are adapted to distinct populations, it is hard to make direct comparisons, but these examples illustrate that risk score calculators weigh variables differently, which could lead to divergent associations between risk scores and migraine.

The importance of migraine as a risk factor for CVD was emphasized when the QRISK score, a risk score calculator based on patients in the United Kingdom, was updated in 2017 and migraine was embedded as an additional variable (19). A study of 24 500 American women was conducted, to evaluate whether incorporating information of migraine with aura would improve two other risk score calculators, *Reynolds Risk Score* and the *ACC/AHA Pooled Cohort Equation*. The fit of the models improved significantly, but the changes were too small to be considered clinically relevant (20).

One key mechanism of migraine aura is cortical spreading depression, which is a slowly spreading wave of depolarization of cortical neurons and glial cells (21). After cortical spreading depression, a short period of hyperemia and thereafter 1–2 h of oligemia follows (22). The reduced blood flow may result in ischemia in tissues with underlying genetic or metabolic susceptibility. Genetic studies have shown loci that predispose for both migraine and stroke (23). These mechanisms are not reflected in SCORE2, because they do not affect sex, age, lipid levels, smoking status or blood pressure. This may partly explain why people with migraine in our study did not have significantly higher SCORE2.

Other studies have examined metabolic syndrome and smoking in relation to migraine. A systematic review from 2019 concluded that the published studies about the potential connection between migraine and metabolic syndrome had weaknesses and that no component of the metabolic syndrome was consistently related to migraine (24). Previous cross-sectional studies from different countries have shown conflicting results regarding the potential association between smoking and migraine (15, 25–27). The only longitudinal prospective study on that topic among adults showed a slightly higher risk of migraine among women who were former or current smokers (men were not included in the study) (28). The inconsistency may contribute to the absence of statistically significant associations in our results.

### 4.1 Strengths and limitations

Our study has several strengths. All 40- and 50-year-olds in a diverse geographic area were invited. The participants were relatively young, which is suitable for studies aiming to improve preventive care, where risk score calculators are most useful. Even though participants were mostly healthy, we present objective measurements of their cardiovascular risk. In contrast to the study by Al-Hassany et al. (6), we present ORs that take both age, sex, level of education and country of birth into account at the same time (instead of only stratifying separately for age and sex).

The main limitation is the register-based migraine diagnoses, which may have led to certain bias. In addition, we could not distinguish between active and past migraine. Consequently, migraine diagnoses accumulated over time, which may partly explain the slightly higher prevalence among 50-year-olds than 40-year-olds (11.2% vs. 10.2%). This distinction is clinically relevant, as the association between migraine and cardiovascular disease appears stronger in individuals with active migraine, while migraine prevalence peaks around 40–44 years of age (3). In most cases we were also unable to distinguish migraine with aura from migraine without aura (29). Since these groups were analyzed together and the cardiovascular connection is stronger for migraine with aura, it is possible that true effects of migraine on SCORE2 were diluted and yielded non-significant results in the entire sample. Another implication of the diagnoses being register-based is that we could not verify that the diagnoses were accurate. However, diagnoses from health care registers are in most cases based on clinical assessments by health care professionals. Given that not all individuals with migraine seek health care, it is likely that some participants with migraine were misclassified as not having migraine. Conversely, diagnostic overlap with other headache disorders, such as tension-type headache, may have led to misclassification among participants diagnosed with migraine. Another limitation is the low number of participants with moderate/high SCORE2 and migraine diagnoses. This led to few cases with the combination of both and might have led to unstable estimates when adjusting for potential confounders. Calculating ORs using logistic regression with the traditional maximum likelihood estimation may cause unreliable estimates and wide confidence intervals (30). To estimate more stable parameters, we chose to use Firth’s penalized models (31).

Preventive medication against migraine is another potential bias. In Scania, the guidelines for migraine state that patients with migraine more frequently than 3 times per month are eligible for preventive treatment. The first line therapies are betablockers, angiotensin receptor blockers and tricyclic antidepressants (32). The two first mentioned therapies lower blood pressure and therefore reduce SCORE2. Unfortunately, we did not have data about the participants’ medications, so our models could not be adjusted for pharmacotherapies. A national Swedish observational study found that during 2019–2021 19% of people diagnosed with migraine used beta-blockers and 3.6% used angiotensin receptor blockers. That study only included migraine diagnoses registered in hospitals, which might have increased the proportion with preventive treatment, since patients only treated in primary care probably have milder migraine (33). However, a study from the UK including patients in primary care presents similar numbers, 18% using beta-blockers and 0.88% using angiotensin receptor blockers (34).

Worldwide, 63% of people with migraine are women (3). In our study, the sex distribution among people with migraine was 79% women, probably because more women (56%) than men accepted the invitation to their THD and because women are more prone to have the diagnosis of migraine confirmed by a health care professional (35), increasing the proportion of women with migraine in this study. Another potential limitation is that the performance of SCORE2 has not been established in migrant populations with diverse ethnic and socioeconomic backgrounds.

### 4.2 Clinical implications

Since previous studies have found that migraine is a risk factor for CVD (1), the results of this study imply that SCORE2 alone might be misleading when assessing cardiovascular risk for patients with migraine. However, given the limitations mentioned above, more studies are needed before the results of this study should impact clinical decision-making. Clinicians need to keep their holistic approach and not solely trust the results of SCORE2 when assessing cardiovascular risk.

## 5 Conclusions

In this cohort, the OR of medium/high SCORE2 was not significantly different between individuals with and without a register-based diagnosis of migraine. That does not necessarily rule out a true association. Future studies should include samples that allow for separate analyses of current and past migraine as well as migraine with and without aura.

## Data Availability

The datasets presented in this article are not readily available because of research ethics regulations. Requests to access the datasets should be directed to the corresponding author. Requests to access the datasets should be directed to elsa.regnell@med.lu.se.
