# Supplementary material for: The association between migraine and SCORE2 - a Swedish cross-sectional study
Source: Front Cardiovasc Med. 2026 Oct 8;13:1854455. doi: 10.3389/fcvm.2026.1854455 (PMC13686835; doi:10.3389/fcvm.2026.1854455)
Supplement: Supplementary file 1 [file Datasheet1.pdf]

Supplementary Table 1. Number of participants in each category of Firth's logistic regression in Table 3.

		Women		Men	
**Model**	**Migraine status**	**Low SCORE2**	**Moderate/high SCORE2**	**Low SCORE2**	**Moderate/high SCORE2**
1	Migraine	675	5	141	46
	No migraine	4301	58	3008	874
2	Migraine	675	5	141	46
	No migraine	4301	58	3008	874
3	Migraine	675	5	141	46
	No migraine	4287	57	3005	873
4	Migraine	675	5	141	46
	No migraine	4284	57	3002	872

Supplementary Table 2. Subgroup analysis based on Firth's penalized logistic regression. Odds ratios for people with migraine with aura to have moderate/high SCORE2 with 95% confidence intervals, compared to people without migraine.
Model 1: Unadjusted. Model 2: Adjusted for age. Model 3: Adjusted for age and education.
Model 4: Adjusted for age, education and country of birth.

**Sex**	**Model**	**OR (CI)**
Women	1	1.81 (0.37-5.38)
	2	1.82 (0.37-5.40)
	3	1.78 (0.36-5.33)
	4	1.94 (0.39-5.87)
Men	1	1.27 (0.59-2.5)
	2	1.27 (0.60-2.52)
	3	1.28 (0.59-2.54)
	4	1.4 (0.65-2.80)

Supplementary Table 3. Number of participants in each category of the subgroup analysis with Firth's logistic regression.

		Women		Men	
**Model**	**Migraine status**	**Low SCORE2**	**Moderate/high SCORE2**	**Low SCORE2**	**Moderate/high SCORE2**
1	Migraine with aura	101	2	28	10
	No migraine	4301	58	3008	874
2	Migraine with aura	101	2	28	10
	No migraine	4301	58	3008	874
3	Migraine with aura	101	2	28	10
	No migraine	4287	57	3005	873
4	Migraine with aura	101	2	28	10
	No migraine	4284	57	3002	872

Supplementary Table 4. Number of participants in each category of linear regression models in Table 4.

Model	Migraine status	Women	Men
1	Migraine	680	187
	No migraine	4359	3882
2	Migraine	680	187
	No migraine	4359	3882
3	Migraine	680	187
	No migraine	4344	3878
4	Migraine	680	187
	No migraine	4341	3874

Supplementary Table 5. Subgroup analysis based on linear regression models. SCORE2 based on migraine with aura, compared with no migraine.

Model 1: Unadjusted. Model 2: Adjusted for age. Model 3: Adjusted for age and education. Model 4: Adjusted for age, education and country of birth.

Sex	Model	Coefficient*	p.value*	Adjusted R^2
Women	1	0.037	0.54	<0.001
	2	0.053	0.26	0.39
	3	0.045	0.33	0.42
	4	0.048	0.29	0.42
Men	1	0.15	0.09	<0.001
	2	0.045	0.55	0.29
	3	0.045	0.54	0.31
	4	0.065	0.38	0.32

* For migraine with aura variable.

Supplementary Table 6. Number of participants in each category of supplementary Table 5.

Model	Migraine status	Women	Men
1	Migraine with aura	103	38
	No migraine	4359	3882
2	Migraine with aura	103	38
	No migraine	4359	3882
3	Migraine with aura	103	38
	No migraine	4344	3878
4	Migraine with aura	103	38
	No migraine	4341	3874
